# Returning to work after treatment for a low-grade glioma: analysing return-to-work rates and exploring barriers experienced by patients

**DOI:** 10.1007/s11060-026-05582-8

**Published:** 2026-04-21

**Authors:** Jasmine C. Kennedy, Stephen J. Price, Tom Manly, Emma Woodberry, Mary Burton

**Affiliations:** 1 Cambridge Brain Tumour Imaging Laboratory, Department of Clinical Neurosciences, University of Cambridge, Cambridge, UK; 2 Cambridge Brain Tumour Imaging Laboratory, Division of Neurosurgery, Department of Clinical Neurosciences, University of Cambridge, Cambridge, UK; 3 MRC Cognition and Brain Sciences Unit, University of Cambridge, 15 Chaucer Road, Cambridge, UK; 4 Neuropsychology Department, Addenbrooke’s Hospital, Cambridge, UK; 5 Astro Brain Tumour Fund, Holme-Next-the-Sea, Hunstanton, Norfolk, UK

**Keywords:** Low-grade glioma, Return to work, Brain tumour, Cognitive rehabilitation, Vocational rehabilitation

## Abstract

**Purpose:**

Low-grade glioma (LGG) brain tumours negatively affect a patient’s quality of life. LGGs typically develop in adults of working age and are incurable. With life expectancy now exceeding 15 years from diagnosis, the clinical focus should be on preserving patients’ quality of life (QoL). Returning to work after oncology treatment has been shown to enhance QoL in cancer patients. However, research specifically exploring LGG patients’ experiences of returning to work is limited. This study examined the return-to-work rate among a sample of LGG patients and identified barriers to return to work.

**Method:**

A total of 47 LGG patients (aged 22–59 years; 31 females, 16 males) responded to an online questionnaire distributed via charity e-newsletters. Data collected included demographics, employment status, and evaluations of clinical services (e.g., vocational rehabilitation). Participants reported their perceived levels of higher cognitive functioning (such as working memory, attention, and decision-making) now compared to before their diagnosis, using a 15-item four-point Likert scale.

**Results:**

Results indicated that over a third of participants (40.4%) had not returned to work, with the majority (68.4%) expressing a desire to do so. Only seven participants received vocational rehabilitation. Symptoms of LGG and travelling to the workplace were identified as barriers to returning to work. Scores for perceived deficits in working memory were significantly higher in participants who had not returned to work (*p* = .027).

**Conclusion:**

The findings emphasise the need for improved clinical services, particularly cognitive assessments and vocational rehabilitation, to support successful return to work for LGG patients.

## Introduction

Gliomas are malignant primary brain tumours arising from mutated glial cells, such as astrocytes and oligodendrocytes [1]. Low-grade gliomas (LGGs) (classified by the World Health Organisation as grade 2 and 3) are slow-growing and characterised by the presence of an IDH gene mutation (astrocytoma) alone, or an IDH mutation and 1p/19q codeletion (oligodendroglioma) [2]. LGGs occur early in an adult’s working life and affect their ability to function in the workplace. Patients can experience a wide range of symptoms and impairments, often co-occurring, with some being more generally cancer-related (e.g., fatigue, pain) while others are more tumour-specific (e.g., seizures, cognitive deficits) [3]. LGGs diffuse into healthy brain tissue, disrupting vital brain networks and structures. LGGs that develop in the pre-frontal cortex can affect higher cognitive functions. Higher cognitive functions are essential cognitive skills for carrying out goal-directed behaviour and include executive functions (working memory, planning and decision-making, task initiation, and cognitive flexibility). These cognitive skills are often intertwined, and challenges in one subskill can affect others. Although incurable, the prognosis for LGG is relatively good, with life expectancy exceeding 15 years from diagnosis. With this increased survival, clinical focus has shifted from being solely treatment-oriented to preserving patients’ quality of life (QoL) after completing oncology treatment [4, 5].

Returning to work has been shown to improve QoL in cancer patients by providing financial stability and social interaction [6]. Unemployment can contribute to financial toxicity and, in the US, can be tied to medical health insurance. Patients described how the loss of identity and purpose at work can have a profound emotional impact, undermining their self-esteem and hope for the future [7]. Not all LGG patients return to work, and the reasons for this remain unclear. In cancer patients, excluding those with brain tumours, neuropsychological function is a predictor of return to work [8]. LGG patients are at a considerable risk of cognitive impairment arising from the tumour, surgery, seizures and adjuvant oncological treatment. Executive functions, in particular, have been shown to be the only cognitive function not to return to baseline levels after LGG surgery [9] and continue to decline three years post-surgery [10]. A retrospective study on LGG patients in Sweden reported that 52% return to work 1 year post-surgery, and this increased to 63% by year 2 [11]. This suggests that over a third of LGG patients don’t return to work by year 2.

Questions have been raised about whether executive function could influence an LGG patient’s ability to return to work successfully. Notably, there are no formal cognitive screening methods available for patients after LGG surgery, creating uncertainty about their executive functioning. Additionally, cognitive and vocational rehabilitation are not standard practices in glioma treatment, and studies using cognitive rehabilitation in stroke patients have failed to rehabilitate executive functions [12]. Moreover, the UK Government defines vocational rehabilitation as “whatever helps someone with a health problem to stay at, return to and remain in work.” [13] However, NHS treatment plans do not include vocational rehabilitation, and return-to-work is not an outcome measure; therefore, the return-to-work rates among UK LGG patients remain unknown, and their specific challenges during this process are unclear.

Qualitative studies among brain tumour patients have highlighted the need for interventions to improve communication between employers and employees, thereby increasing knowledge of return-to-work options [14, 15]. In this study, we investigated the return-to-work rate among our LGG patient group using a questionnaire. We aimed to understand the barriers they face in returning to work after diagnosis and/or completion of oncological treatment by asking open-ended questions. We focused on how their current cognitive functioning, especially executive functions, might influence the experience of returning to work. Additionally, we assessed the effectiveness of clinical services, such as vocational rehabilitation, in supporting return to work in LGG patients.

## Method

### Design

This study employed a cross-sectional mixed method design in the form of a questionnaire. The questionnaire included a mix of quantitative and qualitative questions, which were reviewed and approved by Astro Brain Tumour Fund (https://www.astrofund.org.uk/about-us/), Brainstrust charity (https://brainstrust.org.uk/), and the International Brain Tumour Alliance (IBTA) (https://theibta.org/). This study was approved by the East of England Research Ethics Committee (reference 24/EE/0179) and was conducted in accordance with the Declaration of Helsinki.

### Participants

Individuals were eligible if they were aged ≥ 18 and ≤ 66 years old and diagnosed with an LGG. Purposive and convenience sampling methods were used. Participants were affiliated with Astro Brain Tumour Fund, Brainstrust charity and/or IBTA, respectively, and provided consent to complete the form.

### Materials

A draft questionnaire was created by the researchers and a dedicated Patient and Public Involvement group. This was reviewed and amended by the aforementioned organisations, and the final questionnaire was approved and implemented in Google Forms, with links provided to Astro Brain Tumour Fund, Brainstrust, and IBTA. The questionnaire consisted of 54 questions. The quantitative response options included yes/no, multiple-choice, checkbox, and Likert scale formats. The qualitative response options included short- and long-answer text boxes. Participants were informed that their responses would remain anonymous. The questionnaire was divided into sections, which were as follows:

1. Questionnaire Introduction.
2. Participant demographics.
3. Participants who had returned to work completed this section. The questions aimed to understand whether any aspects of their work had changed since returning to work, or whether they had encountered any issues with their return.
4. Participants who had not returned to work or were on sick leave completed this section. The questions aimed to explore their reasons for not returning to work.
5. All participants completed this section. Questions were asked about whether the participant experienced fatigue and seizures. Participants were asked to rate their perceived levels of higher cognitive functioning now compared to before their diagnosis. These questions were inspired by skills mentioned in the Executive Skills Questionnaire for Adults [16].
6. To conclude, the aim of the questionnaire was again outlined to the participants. Participants were invited to continue the study by providing their email addresses, which would allow them to be contacted with more information later.

### Procedure

Prospective participants were contacted via the Astro Brain Tumour Fund, Brainstrust, and IBTA Facebook support groups and/or e-newsletters and invited to complete the questionnaire. The Facebook post and e-newsletter included a brief description of the study’s purpose, its affiliations, and a link to the Google Form. Data collection occurred over 3 months.

### Data analysis

The qualitative data was used to enrich the quantitative results in the form of quotes, providing context and emphasis. The quantitative data was analysed using the Statistics Package for the Social Sciences (SPSS) [17]. The rates of return to work, non-return to work, and sick leave were assessed among our participants. The results from the higher cognitive function Likert scale were explored in IMB’s Statistical Package for the Social Sciences (SPSS) (Version 27). The 4-point Likert scale consisted of 14 items (α = 0.92), on which participants rated their perceived higher cognitive functioning now compared to before their diagnosis. The maximum score was 60, and the minimum was four. A higher score would indicate a more severe perceived cognitive deficit than pre-diagnosis cognitive function. A lower score would indicate improved cognitive function relative to pre-diagnosis levels. The dependent variable was the total Likert scale score. The independent variable was participants’ work status (2 groups: Returned to Work and Non-Return to Work. Participants on sick leave were included in the Non-Return to Work group. A Mann-Whitney U test was used to determine whether there was a difference between the two groups’ (Returned to Work vs. Non-Return to Work) total test scores and on the individual Likert-scale questions. The result was deemed statistically significant if *p* ≤ .05.

## Results

### Participant characteristics

A total of 47 LGG patients (aged 22–59 years, 31 females and 16 males) responded to the questionnaire. Table 1 summarises participants’ demographics. 28 participants (59.6%) had returned to work, 15 (31.9%) had not returned to work, and 4 (8.5%) were on sick leave at the time of the study. Seven participants did not take time off work following their LGG diagnosis. 24 participants (51%), self-reported being diagnosed with astrocytoma, 10 (21.3%) with oligodendroglioma, 4 (8.5%) with ependymoma and one (2.1%) with oligoastrocytoma. 19 (40.4%) of our participants underwent surgery, and adjuvant oncology treatment (radiotherapy and chemotherapy), 17 (36.2%) received surgery alone, and 6 (12.8%) were in active surveillance. One participant (2.1%) was pre-surgery, and two (4.3%) were receiving adjuvant oncology treatment at the time of study.

### Return to work

On average, participants returned to work 15.2 weeks after their diagnosis and/or after completing oncological treatment. 21 participants (75%) returned to their previous jobs; one participant (3.6%) started a new role within the same organisation; five participants (17.9%) began new jobs in different organisations; and one participant (3.6%) did not specify. Before diagnosis, 21 participants (75%) worked full-time, while four of the 21 participants later changed to part-time contracts after their diagnosis. Regarding workplace arrangements, 11 participants (39.3%) worked on-site (e.g., in an office), six (21.4%) worked remotely (e.g., from home), and 11 (39.3%) had a hybrid setup combining remote and on-site work. Three participants (10.7%) were self-employed. Participant job titles are listed in Table 2. Most participants drove to work (42.3%). Eleven participants (39.3%) reported that their mode of transport changed after diagnosis, and seven participants (25%) reported that travelling to work was an issue. “*Driving became a huge issue when I lost my licence as I didn’t live on a transport link route. Work didn’t really offer much in terms of support. I have had to rely on asking for lifts everyday for the last 9 months which has been hell organising on top of my job*,* as well as childcare/school pickups*, etc.” (Pa8, 40, female, unknown tumour type, family engagement manager).

### Workplace adjustments

13 (46.4%) participants experienced changes to their working hours, nine (32.1%) participants faced modifications to their workplace setting, eight (28.6%) participants had adjustments to their workload, and six (21.4%) participants saw changes to their work tasks. “*I worked from home back in 2013*,* then went out with an engineer until I got my license back”* (Pa32, 36, male, unknown tumour type, field line manager). Nine participants (32.1%) reported that their employer made no workplace adjustments. Most of the 13 participants (78.6%) stated that they did not require any further workplace modifications.

**Table 1:** Participant characteristics

Characteristic	Return to Work		Non-Return to Work		Sick leave	
	*n*	%	*n*	%	*n*	%
**Gender**						
Female	18	64.3	10	66.7	3	75
Male	10	35.7	5	33.3	1	25
**Country of Residence**						
Australia			1	6.7		
Bangladesh	1	3.6				
Canada			1	6.7		
Japan	1	3.6	1	6.7		
Netherlands	1	3.6	1	6.7		
United Kingdom	22	78.6	11	73.3	4	100
USA	3	10.7				
**Ethnicity**						
Asian/Asian British	4	14.3	1	6.7		
White	24	85.7	14	93.3	4	100
**Highest level of Education**						
No formal qualifications	1	3.6				
GCSE	2	7.2	3	20		
A-Levels	2	7.2	3	20	1	25
Apprenticeship	1	3.6				
Diploma	2	7.2	2	13.3		
Degree Level	13	46.4	5	33.3	2	50
Higher Level	7	25	2	13.3	1	25
**Patient Reported Tumour Type**						
Astrocytoma	14	50	8	53.3	2	50
Ependymoma	3	10.7			1	25
Oligodendroglioma	4	14.3	6	40		
Oligoastrocytoma	1	3.6				
Unknown	6	21.4	1	6.7	1	25
**Treatment Type/Stage**						
Active surveillance	5	17.9	1	6.7		
Waiting for surgery	1	3.6				
Beginning/going through oncology treatment	1	3.6			1	25
Oncology treatment only	1	3.6	3	20		
Surgery only	8	28.6	5	33.3	3	75
Surgery and active surveillance	1	3.6				
Surgery and oncology treatment	9	32.1	5	33.3		
Surgery, oncology treatment and active surveillance			1	6.7		
Other	1	3.6				
Unknown	1	3.6				

Note. *N* = 47. Oncology treatments included chemotherapy, radiotherapy and proton-beam therapy. The treatment stage answer which fell under ‘other’ was a phase three clinical trial

### Motivations for returning to work

18 (64.3%) participants who had returned expressed a desire to return to work, with job satisfaction motivating 13 (46.4%) of them. However, 13 (46.4%) participants cited financial pressure as a reason for returning to work, while two (7.1%) participants felt societal or familial pressure to return. “*Enjoy the intellectual stimulation of what I do*,* and having a good structure to my week. Financially contributing and in an industry I find super interesting****”*** (Pa34, 39, female, oligodendroglioma, business development/marketing).

### Quality of life

**Fig. 1:**
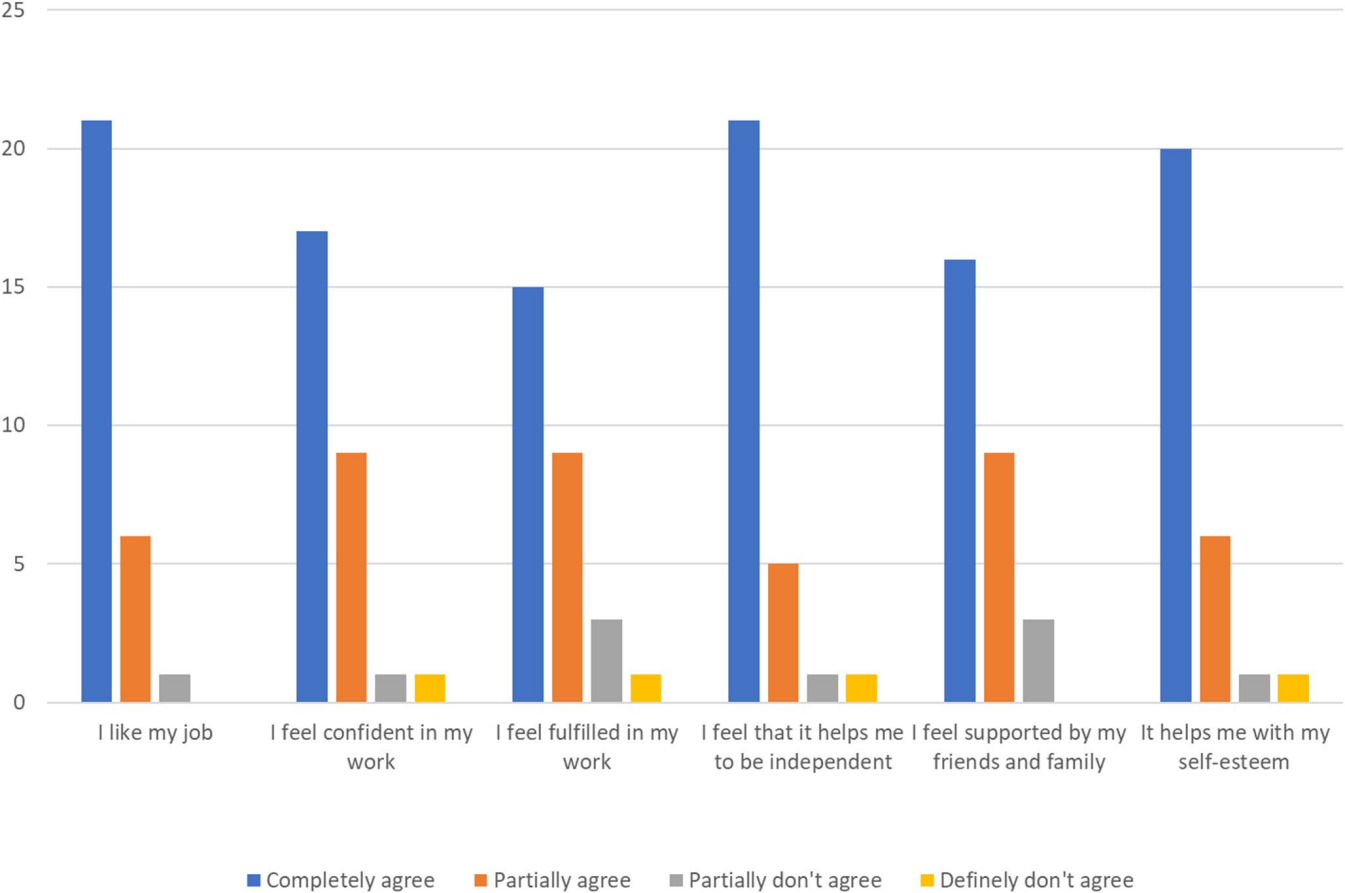
Bar chart displaying participants’ answers on a 4-point Likert scale

Participants were asked to rate their level of agreement (completely agree = 4, partially agree = 3, partially do not agree = 2, definitely do not agree = 1) to six statements on a 4-point Likert scale; I like my job (M = 3.64, median = 4), I feel confident in my work (M = 3.45, median = 4), I feel fulfilled in my work (M = 3.27, median = 3.5), I feel that it helps me to be independent (M = 3.55, median = 4), I feel supported by my family and friends (M = 3.36, median = 3.5), and it helps with my self-esteem (M = 3.55, median = 4). The results are displayed as a bar chart in Fig. 1. “*My work doesn’t feel like work*,* I love and enjoy what I do otherwise I wouldn’t do it. And that’s with everything I do in general in life”* (Pa28, 22, male, astrocytoma, personal trainer).

**Table 2:** Participant job titles and work status at time of study

Returned to work	Non-return to work	Sick leave
Administrator	Administration	Carer
Archaeologist	Broadcast technical manager	Optometrist
Business development and marketing manager	Injection moulding machine operative	Shipping manager
Business support	Investor	Veterinary surgeon
Chartered surveyor	Lawyer	
Children services manager	NA - GCSE student	
Data analyst	NA - Student	
Dog walker	NHS	
Family engagement manager	NHS carer	
Field line manager	Optometrist	
First aider	Retail manager	
House manager	Secondary school teacher	
Front of house and conference services operations manager	Self-employed in education	
IT auditor	Senior manager in private care	
Kindergarten director	Tax accountant	
Lawyer		
Managing director		
Mental health practitioner		
Molecular biologist		
Molecular biologist		
Operations director		
Personal trainer		
Physician assistant		
Playworker		
Research officer		
Senior UX & UI designer		
Shutdown planning lead		
Urgent care physician		

### Non-return to work

Of the 19 participants (68.4%) who had not returned to work or were on sick leave, most expressed a desire to return. Over half (54.5%) of the 13 participants who wished to return to work reported feeling pressured to do so. Types of pressure included financial pressure (90%), employer pressure (40%), family or societal pressure (60%), and personal pressure (70%). Thirteen (68.4%) participants indicated that their LGG symptoms prevented them from returning to work. The outcomes of surgery and treatment also led 13 (68.4%) participants to cease working. The need to travel to a workplace was an issue for 12 (63.2%) participants. The majority of the 19 (63.2%) participants felt their employers were not supportive in facilitating their return to work. However, they felt supported by family and friends (89.5%). One participant (5.3%) in the non-return to work group was self-employed.

### Clinical services

All participants were asked whether they had received vocational rehabilitation. Only seven (14.9%) participants received vocational rehabilitation after completing treatment for their LGG; three were very satisfied, four were satisfied, and one was very dissatisfied with this service. Several participants (40.4%) expressed interest in receiving vocational rehabilitation. Of those who received vocational rehabilitation, three (42.6%) returned to work, three (42.6%) did not, and one (14.3%) was on sick leave.

### Fatigue and seizures

All participants were asked whether they experienced fatigue and seizures. Most participants (57.4%) reported experiencing fatigue often. “*Fatigue affects my day to day”* (Pa9, 29, female, astrocytoma, business support). Of these participants, 13 (59.1%) were working, six (27.3%) were not working, and three (13.7%) were on sick leave. Eight participants (17%) reported always feeling fatigued; of these, four were working and four were not. 34 participants (72.3%) were on medication for seizures. 20 (58.9%) of these participants were back at work, ten participants (29.4%) were not at work, and four participants (11.8%) were on sick leave.

### Higher cognitive functions and return to work

The dataset contained two outliers, as indicated by the boxplot. A Mann-Whitney U test was used to compare the mean ranks of the total scores of each group (Returned to Work and Non-Return to Work) and on individual questions. The assumptions of a Mann-Whitney U test were met in our data; the dependent variable was ordinal, the independent variable had two categorical independent groups (Returned to Work vs. Non-Return to Work), the observations were independent, and the data were not normally distributed.

Although the Non-Return to Work group had the highest mean rank of total scores in the self-reported higher cognitive function deficit questions, a Mann-Whitney U test revealed no significant difference in self-reported deficits in higher cognitive function now in comparison to pre-diagnosis in LGG participants who had returned to work (Returned to Work - Median = 34.50, *n* = 28) vs. those not returned to work (Non-Return to Work - Median = 37, *n* = 19, Mann-Whitney *U-test* = 207, *P* = .200). The total scores were higher in the Returned to Work group than the Non-Return to Work group (935 vs. 706, respectively).

The average scores for perceived deficits in higher cognitive function, compared with at the time of diagnosis, were lower in participants who had returned to work than those who had not returned to work (*M* = 33.39, *SD* = 5.928 vs. 33.39, 95% CI:) or were on sick leave (*M* = 37.18, *SD* = 10.112). The effect size for the difference between the groups was calculated using Cohen’s d, *r* = .19, which is considered a small effect. The Non-Return to Work group had a significantly higher test score in Question 1 (“How would you rate your memory now for things you should do?”), than the Returned to Work group, *U* = 172.50, *p* = .027. “*Memory and speech problems. Lack of confidence”* (Pa33, 55, female, ependymoma, molecular biologist).

## Discussion

In this cross-sectional mixed method study, we aimed to calculate the return-to-work rate in our LGG sample and understand their experience of returning to work. The successful return-to-work rate among our sample was 59.6%. This result highlights that, at the time of the study, over a third of participants had not returned to work after diagnosis or completion of oncological treatment (15 (31.9%) had not returned to work, and four (8.5%) were on sick leave). Only seven of the 47 participants received vocational rehabilitation. Symptoms of LGG and travelling to the workplace were identified as barriers to returning to work. Participants in both the Returned to Work and Non-Return to Work groups self-reported cognitive deficits. Working memory was reported to be worse in the Non-Return to Work group. Fatigue was a commonly reported issue amongst our participants who had returned to work.

The return-to-work rate in our LGG patient group is consistent with previous studies. Similarly, our findings are in keeping with research that neuropsychological function predicts return to work in cancer patients as LGG patients who did not return to work reported greater deficits in executive functions than those who had returned to work. Working memory was a concern reported by patients who had not returned to work and is a vital cognitive function for employment. Working memory is the capacity to retain small amounts of information in mind and is heavily involved in goal-directed behaviours that require information to be maintained and manipulated to ensure successful task execution [18]. Many of our participants who had returned to work reported often experiencing fatigue. Research indicates that both mental and physical fatigue frequently occur in LGG and are linked to long-term return-to-work outcomes [19, 20].

Returning to work after treatment for a brain tumour is important for QoL and financial security, but it is not an easy feat, and participants reported difficulties during their working day. However, those who had returned to work reported job satisfaction and high self-esteem. Our results emphasise the crucial role of cognitive function in successful return to work and expose the extent of cognitive impairment experienced by LGG patients. The findings highlight weaknesses in the standard of care provided by the NHS and the need for further research and clinical advancements to support return to work in LGG patients. Additionally, fatigue management should be incorporated into the NHS treatment plan to improve return-to-work outcomes.

There were several limitations to our study. The questionnaire did not capture participants’ treatment stage, so it is unclear how many were still in treatment or had completed treatment. We did not capture information on relationship status or dependents. This could affect participants’ motivation for returning to work and may vary more across age groups. Participants were recruited through the social channels of Astro Brain Tumour Fund, brainstrust, and IBTA, and were self-selected, potentially having more time, interest, and capacity to take part. Those carrying on “as normal”, meaning people who are managing well with a lesser impact, may have been underrepresented or missed. The present study did not analyse full-time versus part-time work contracts, which could be explored in future research. We only collected self-reports of executive function deficits. Some participants may not even be aware that they have deficits.

## Conclusion

This study evaluated the return-to-work rate following a diagnosis of LGG and/or the completion of treatment. Executive functions play a vital role in completing work-related tasks. Return to work in LGG remains poorly understood, and this issue has not received sufficient attention from researchers, clinicians, employers, or government bodies. Future research should examine the impact of deficits in executive function and fatigue on return to work in patients with LGG.

## Data Availability

Data is available for reasonable requests to corresponding author.
